# Implementation and Use of a Client-Facing Web-Based Shared Decision-Making System (MyCHOIS-CommonGround) in Two Specialty Mental Health Clinics

**DOI:** 10.1007/s10597-018-0341-x

**Published:** 2018-10-13

**Authors:** Molly Finnerty, Elizabeth Austin, Qingxian Chen, Deborah Layman, Edith Kealey, Daisy Ng-Mak, Krithika Rajagopalan, Kimberly Hoagwood

**Affiliations:** 1 New York State Office of Mental Health, 330 5th Ave, New York, NY 10001 USA; 2 New York University Langone Medical Center, One Park Ave, 7th Floor, New York, NY USA; 3 New York State Office of Mental Health, 75 New Scotland Ave, CDPC Unit R, Albany, NY USA; 4 Sunovion Pharmaceuticals Inc., 84 Waterford Drive, Marlborough, MA 01752 USA; 5 Present Address: Department of Surgery, University of Washington, 1107 NE 45th Street, Box 354808, Seattle, WA USA; 6 Present Address: NYC Department of Social Services, 150 Greenwich St., 42nd Floor, New York, NY USA

**Keywords:** Shared decision-making, Serious mental illness, Health information technology, Peer support, Implementation

## Abstract

Electronic shared-decision making programs may provide an assistive technology to support physician–patient communication. This mixed methods study examined use of a web-based shared decision-making program (MyCHOIS-CommonGround) by individuals receiving specialty mental health services, and identified qualitative factors influencing adoption during the first 18 months of implementation in two Medicaid mental health clinics. T-tests and χ^2^ analyses were conducted to assess differences in patient use between sites. Approximately 80% of patients in both clinics created a MyCHOIS-CommonGround user profile, but marked differences emerged between clinics in patients completing shared decision-making reports (79% vs. 28%, χ^2^_(1)_ = 109.92, p < .01) and average number of reports (7.20 vs. 3.60, t = − 3.64, p < .01). Results suggest high penetration of computer-based programs in specialty mental health services is possible, but clinic implementation factors can influence patient use including leadership commitment, peer staff funding to support the program, and implementation strategy, most notably integration of the program within routine clinical workflow.

Shared decision-making is a model of physician–patient communication often paired with decision aids that involve both patients and physicians in decision-making processes (Charles et al. 1997). The aim of this communication model is to involve both patients and physicians in decision-making processes by balancing clinical information about health conditions and treatment options with an individual’s preferences, goals, and cultural values. Shared decision-making has been associated with improved treatment adherence, increased patient knowledge, and improved mutual understanding between patient and clinician with the strongest evidence for improved patient satisfaction and reduced decisional conflict (Clayman et al. 2016; Duncan et al. 2010; Eliacin et al. 2014; Joosten et al. 2008; Shay et al. 2015; Simon et al. 2009; Swanson et al. 2007). Physician use of shared decision-making has been promoted by the Institute of Medicine and the U.S. Preventative Medicine Services Task Force (Berwick 2002; Institute of Medicine and Committee on Quality of Health Care in America 2001; Sheridan et al. 2004). However, a review of practice research highlights that clinicians do not consistently involve patients in the decision-making process, particularly in eliciting and incorporating patient preferences (Couët et al. 2015). A national patient survey suggests that clinicians are less likely to engage patients with mental health conditions in clinical decision-making, particularly those with persistent mental illness (Rowan and Shippee 2015).

Health Information Technology (HIT) may support shared decision-making by automating and documenting key elements of decision support, including eliciting patient preferences, concerns, and prioritizations for care (Deegan 2010; Drake et al. 2010; Korsbek and Tønder 2016; Murray et al. 2005; Ruland and Bakken 2002; Woltmann et al. 2011). For individuals with serious mental illness, HIT-based shared decision-making tools may also provide an assistive technology to support physician–patient communication (Deegan et al. 2008). Research on computer-based resources and interventions suggests that individuals with serious mental illness can effectively use technology in treatment (Ben-Zeev et al. 2013; Druss et al. 2014; Korsbek and Tønder 2016; Schrank et al. 2010; van der Krieke et al. 2012, 2014). However, there is little or no published data on the penetration of applications designed for individuals with serious mental illness, specifically the proportion of individuals receiving mental health services who become users when it is implemented in their service setting (Bonfils et al. 2016; Deegan et al. 2008, 2017; Deegan 2010; Druss et al. 2014; Goscha and Rapp 2015; Kipping et al. 2016; Korsbek and Tønder 2016; Salyers et al. 2017; Stein et al. 2013; van der Krieke et al. 2014). Investigators have previously reported on consumer use of CommonGround, a shared decision-making application for individuals with serious mental illness, but penetration data were not available (Bonfils et al. 2016; Campbell et al. 2014; Deegan 2010; Deegan et al. 2017; Goscha 2009; Salyers et al. 2017; Stein et al. 2010). Information on penetration and use of computer-based applications by individuals with serious mental illness is critical for understanding the feasibility and potential impact of these interventions for these individuals in real-world settings.

The current study examined the use and penetration of MyCHOIS, an electronic consumer-facing application that incorporates the CommonGround shared decision-making program. We examine use by individuals receiving services in two New York City-based Medicaid mental health clinics during the first 18 months of program implementation. In addition, we examine the implementation processes in these pilot clinics to explore the relationship between clinic implementation strategies and penetration rates, in preparation for a larger scale implementation.

## Methods

### Web-Based Shared Decision-Making Tool

My Collaborative Health Outcome Information System (MyCHOIS) is the consumer-facing component of the Psychiatric Services and Clinical Knowledge Enhancement System (PSYCKES), a web-based platform for supporting clinical decision-making and quality improvement, which was developed by the New York State (NYS) Office of Mental Health under Dr. Molly Finnerty. CommonGround, a shared decision-making program, is one tool available to consumers in MyCHOIS. CommonGround, developed by Pat Deegan & Associates, Ph.D., LLC (PDA), guides consumers through a questionnaire that results in the CommonGround Health Report, which is a summary of the individual’s treatment goals, preferences, priorities, and outcomes (Deegan 2010). The MyCHOIS-CommonGround Health Report questionnaire has several features designed to support usability, including one question per page, touchscreen navigation with minimal to no keyboard entry required, listen/read options, and English and Spanish language options. In addition, client use of the CommonGround program is supported by peer staff (i.e., individuals with personal experience with the mental health system) who provide “embodiments of recovery”, a person-centered approach, and encourage self-advocacy and responsibility (Austin et al. 2014). Peer staff provide technical support, including registering clients, assisting with login and report completion, printing, and review as needed (Deegan 2010).

### Setting

MyCHOIS-CommonGround was piloted in a convenience sample of two Medicaid mental health clinics in New York City in preparation for a larger scale implementation. The two clinics had a combined census of 543 patients (See Table 1); all clinic patients were used in the analysis. Considerations for selecting pilot clinics included: (1) peer workers on staff (that could be reassigned to the MyCHOIS-CommonGround application), (2) no concurrent HIT implementation, and (3) clinic leadership commitment to fulfilling the implementation protocols, including establishing a peer-staffed kiosk in or near the clinic waiting room. The study protocol was approved by the New York State Office of Mental Health Institutional Review Board located at the Nathan Kline Institute for Psychiatric Research with a waiver of informed consent.

**Table 1:** Characteristics of two MyCHOIS-CommonGround pilot clinics

Pilot clinic characteristics	Clinic 1	Clinic 2
Patient census (total caseload)	409	134
Physicians: number, total FTE^a^	4 (FTE = 4)	2 (FTE = 0.5)
Peer staff: number, total FTE	4–5 (FTE = 2–3)	1 (FTE = 0.5)
Patients with Spanish language preference %	35%	16%
Patients with Medicaid insurance %	63%	65%

*FTE* full time equivalent

### Implementation Protocol

**Fig. 1:**
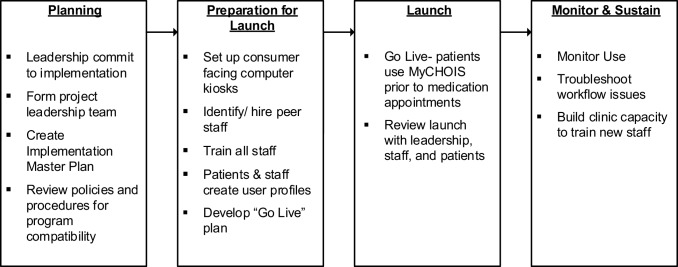
Overview of MyCHOIS-CommonGround implementation phases and milestones

The project Technical Assistance (TA) Team used a four-phase CommonGround implementation protocol developed by Pat Deegan and Associates which included planning, preparation for launch, launch, and monitor and sustaining phases (summarized in Fig. 1). Clinics developed a milestone-based work plan in collaboration with the TA team, identifying staff responsible for each milestone and estimated date of completion. The TA team met a minimum of monthly with clinic project leadership teams throughout the pilot to support and monitor implementation, provide staff training, and obtain feedback on the MyCHOIS-CommonGround program. The implementation goal was full integration of the CommonGround shared decision-making program in clinic workflow.

### Measures of Use and Data Analysis

MyCHOIS-CommonGround use logs were extracted to assess three measures of use and penetration of the CommonGround program by clinic patients: creation of a user profile, completion of one or more CommonGround shared decision-making reports, and average number of reports (among those completing one or more).

#### Creation of User Profiles

The proportion of clinic patients who had completed a user profile as of the last day of the measurement month, including the individual’s overarching treatment goal (“Power Statement”), personal wellness activities (“Personal Medicine”); and warning signs of relapse.

#### Completion of a CommonGround Shared Decision-Making Report

The proportion of current clinic clients who had completed one or more shared decision-making reports as of the end of the period of observation.

#### Average Number of CommonGround Shared Decision-Making Reports Among Users

The number of shared decision-making reports completed as of the end of the period of observation (18 months) among those who had completed one or more reports.

Differences in patient use of the application between clinics were examined using Chi-Square and t-tests.

### Qualitative Assessment and Analysis of Implementation Processes

A qualitative assessment of the pilot implementation was conducted to understand the relationship between implementation experiences at each clinic and client use of the program. Qualitative data sources included all implementation documents developed by the implementation team or pilot clinics during the project. Notes were systematically recorded by the implementation team during clinic visits and meetings, including pilot site implementation meetings, site visits, clinic staff trainings, usability testing, and user feedback sessions. Additional sources included clinics’ implementation plans submitted to the implementation team, and implementation team impressions.

We used a mixed methods approach similar to O’Cathain’s methodological description of “following a thread” (Moran-Ellis 2006; O’Cathain et al. 2010) to understand the relationship between quantitative measures of client use and clinic implementation strategies and challenges over time. Qualitative data were analyzed using a constructivist approach (Creswell 2012), and summarized under five domains based on the Consolidation Framework for Implementation Research (CFIR): intervention characteristics, inner setting, outer setting, characteristics of individuals, and implementation process (Damschroder et al. 2009). All authors certify responsibility for the manuscript.

## Results

### Creation of a MyCHOIS-CommonGround User Profile by Clinic Patients

**Fig. 2:**
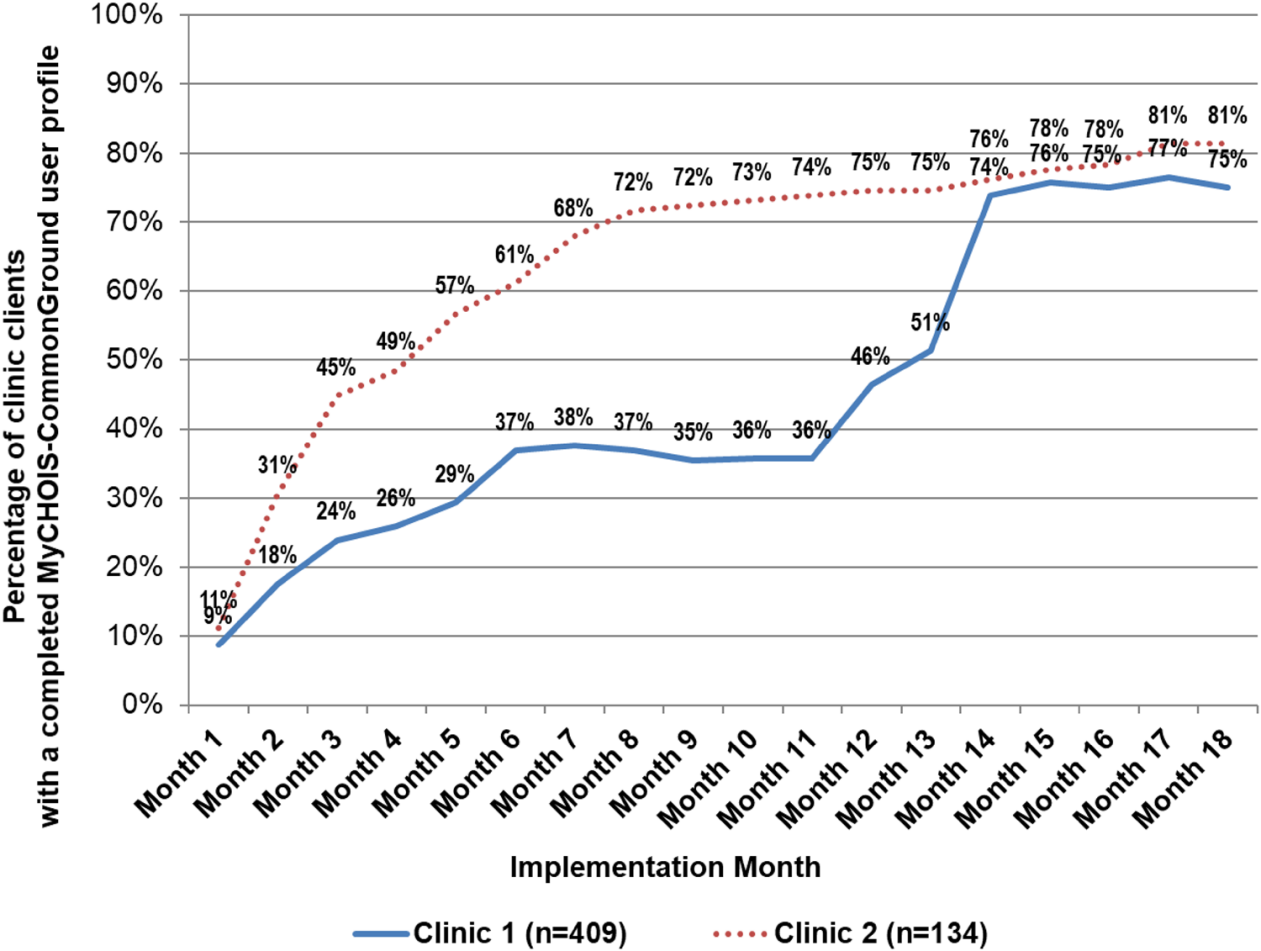
Percent of clinic patients who created a MyCHOIS-CommonGround user profile

The proportion of clinic patients who created a MyCHOIS-CommonGround user profile, including their treatment goal, personal wellness activities, and early signs of relapse, was 77% (n = 416) at 18 months (see Table 1). Figure 2 presents the proportion of clinic patients who had completed a MyCHOIS-CommonGround user profile by the end of each month over the 18-month period of observation.

### Completion of MyCHOIS-CommonGround Shared Decision-Making Reports

Over the 18-month period of observation, 41% of clinic patients used MyCHOIS-CommonGround to create a CommonGround shared decision-making report prior to an appointment with their physician (See Table 2). Penetration at Clinic 2 was significantly higher than Clinic 1, with over 79% of Clinic 2 patients using the shared decision-making application one or more times over the period of observation (vs. 28% in clinic 1; Chi-Square = 109.92 *p* < .01). In addition, patients at Clinic 2 used the shared decision-making application more often than patients at Clinic 1, with a higher average number of shared decision-making reports per user over the first 18 months (7.18 vs. 3.61 average reports/user, t = − 3.64, p < .01).

**Table 2:** Use of the MyCHOIS-CommonGround shared decision-making application by clinic patients

Measures of MyCHOIS use by clinic patients 0–18 months	Total N = 543	Clinic 1 N = 409	Clinic 2 N = 134	Clinic 1 vs. Clinic 2
Creation of a user profile	77% (n = 416)	75% (n = 307)	81% (n = 109)	ns
Completion of a SDM report	41% (n = 220)	28% (n = 114)	79% (n = 106)	χ^2^_(1)_ = 109.92 *p* < .01
Number of SDM reports completed	983	356	627	
Range of SDM reports completed	0–17	0–16	0–17	
Average (Standard deviation) SDM reports completed (among patients completing one or more)	5.33 (7.62)	3.61 (9.03)	7.18 (5.16)	t = − 3.64, *p* < .01

*SDM* shared decision-making

### MyCHOIS-CommonGround Implementation: Qualitative Findings

Qualitative findings explore differences in the two clinics’ implementation strategies and challenges which provide context to patient utilization outcomes at each site. Results are summarized by the five CFIR domains below: inner setting, outer setting, characteristics of individuals, and implementation process (Damschroder et al. 2009).

### Intervention Characteristics

#### Ongoing Application Development

The MyCHOIS-CommonGround application continued to be developed, based in part on feedback from the clinics, throughout the pilot. Leadership responded differently to the challenge of ongoing application development at each clinic. For example, after go-live, Clinic 1 requested enhancements and suspended further implementation until the application was updated, creating a plateau in user profile creation (See Fig. 2), while Clinic 2 continued implementation of MyCHOIS-CommonGround, despite ongoing development.

### Outer Setting: External Policies and Incentives

#### Peer Staff Funding

Peer staff services in clinics and shared decision-making are not funded by Medicaid or commercial insurance in New York State. For example, there is no enhanced reimbursement rate for physician visits that include shared decision-making or direct reimbursement for peer staff supporting the program. Clinics differed in their approach to funding critical peer support staff positions, which impacted the stability of the program and the implementation outcomes at each site. Clinic 1 did not reassign their existing peer staff to the MyCHOIS-CommonGround project and instead worked with the TA team to arrange for temporary peer staffing using two different approaches: (1) contract with a peer services agency, and (2) unpaid peer internships. Although these strategies provided temporary support for the MyCHOIS-CommonGround implementation, it also led to role confusion over who had responsibility for supervision and management of peer staff, and it led to challenges integrating and retaining peer staff at Clinic 1. At Clinic 2, a bilingual peer specialist already on staff was assigned to support the MyCHOIS-CommonGround implementation, which led to greater stability and integration of this critical implementation role. Coverage for the peer staff during vacations and other absences was a challenge for both clinics, and the program was suspended any time peer support staff were absent for any reason, which created disruptions in program workflow routines.

### Inner Setting: Organization’s Structural and Cultural Context

#### Engagement of Local IT Staff

The lack of strategic engagement of IT staff created a challenge at Clinic 1, which is part of a large hospital system, but not at Clinic 2, a smaller clinic within a smaller provider agency. For example, the TA team ordered requested IT equipment for Clinic 1, with the unintended consequence of circumventing usual hospital protocols and supports. Consequently, installation of equipment was chaotic, and after go-live, the MyCHOIS-CommonGround program had to be temporarily suspended while patient computers were removed for reimaging and reinstalled. At Clinic 2, part of a smaller agency, computer installation and setup was managed locally by the clinic director and an on-site IT support staff; minimal TA team involvement or assistance was required.

#### Local Leadership Autonomy and Commitment

The clinic leadership decision to implement the MyCHOIS-CommonGround shared decision-making program is a critical first step in the implementation process. The clinic director at Clinic 2 had the autonomy to make the implementation decision and was committed to the program’s success. For example, the Clinic 2 director gave up her office near the waiting room for MyCHOIS-CommonGround computer kiosks and peer staff and communicated an expectation for full integration into clinic workflows to all staff and clinic patients.

At Clinic 1, the hospital Executive Director made the implementation decision, and both the hospital Executive Director and the Clinic Director retired prior to program launch. The new Clinic 1 director took a more cautious implementation approach and protected clinic staff time and resources by starting with just one physician in the clinic, asking the TA team to find additional staff to support the program rather than dedicating their existing peer staff, and diverting tasks to the TA team and TA team-funded peer staff.

### Characteristics of Individuals: Users Involved in the Implementation

#### Shared Decision-Making Attitudes

In both clinics, physicians initially were ambivalent about the need for a program that supported shared decision-making. In general, they believed that they were already using a shared decision-making approach, had good communication with their patients, and knew their patients’ goals, concerns, preferences, and outcomes. They were also concerned the program would require additional time and effort. It was only in Clinic 2, where penetration of MyCHOIS-CommonGround was high, that a physician reported that his attitude shifted over time with use of the CommonGround shared decision-making reports. The physician noted that he came to depend upon the shared decision-making reports that patients completed, and he believed that these reports added both clinical value and efficiency to his appointments. If a patient came to his appointment without having first completed a report, he would walk them back to the MyCHOIS-CommonGround computer kiosk to complete a report with peer support.

### Implementation Process: Change Process

#### Embedding in Clinic Workflow

The clinics’ ability to embed MyCHOIS-CommonGround user profile creation and program use into the clinical workflow had a notable impact on measures of patient use. In Clinic 2, user profile creation was integrated into routine therapy appointments; the patient and therapist worked together to create the MyCHOIS-CommonGround user profile during one of their usually scheduled sessions. In Clinic 1 they assigned peer staff to try to engage patients in the waiting room. This approach led patients to perceive the program as an optional peer service, rather than as an integral part of clinic service, and user profile creation advanced more slowly. At month 12, Clinic 1 adopted the strategy used by Clinic 2 and embedded user profile creation within therapy visits. This action doubled the proportion of patients with completed user profiles in 3 months and achieved a level of penetration comparable to Clinic 2 (see Fig. 2).

The clinical workflow for completion of CommonGround shared decision-making reports also differed between clinics and impacted patient use of the application. Clinic 2 embedded MyCHOIS-CommonGround shared decision-making reports into routine workflow for physician appointments. All patients checked in with the front desk. Peer staff or front desk staff then escorted patients from the waiting room to the MyCHOIS-CommonGround computer kiosks and logged them into the application. Patients completed a shared decision-making report with peer assistance, as needed, and peer staff escorted the patient to the physician’s room. In Clinic 1, there were multiple challenges to establishing a clinical workflow that integrated ongoing use of MyCHOIS-CommonGround for shared decision-making. First, Clinic 1 did not have a systematic patient workflow management process in place to build upon. For example, patients moved freely about the clinic and often bypassed the front desk check-in and waiting room. There was also no centralized scheduling for physician’s appointments, and some physicians used a drop-in scheduling approach. A second challenge was that the program was phased in, so a standard clinical workflow could not be established for all patients during the first 6 months. Finally, there were intermittent pauses over the 18-month period of observation where the shared decision workflow was suspended due to decisions to wait for technology enhancements as well as peer staff turnover.

## Discussion

This paper examines use of MyCHOIS-CommonGround, a web-based shared decision-making program, during the first 18 months of program implementation in two Medicaid mental health clinics. Our results indicate that a high level of penetration of a HIT-based shared decision-making program is possible in this service setting, with approximately 80% of all clinic patients creating a MyCHOIS-CommonGround user profile, personalizing the application with their treatment goal, wellness activities, and early warning signs of relapse. However, there were significant differences between clinics in patient use to create shared decision-making reports, associated with critical differences in the implementation at each site.

This study explores a critical gap in the literature on the penetration of consumer-facing computer applications in routine specialty mental health clinic populations. It builds upon multiple previous reports that have established that individuals with serious mental illness can use technology (Ben-Zeev et al. 2013; Druss et al. 2014; Korsbek and Tønder 2016; Schrank et al. 2010; van der Krieke et al. 2012, 2014), and suggests that not only some, but most individuals can participate in web-based programs in routine mental health treatment settings, with sufficient support. This is important given the increasing interest in the development and evaluation of web-based mental health applications (Bakker et al. 2016; Luxton et al. 2011). Understanding patient perspectives on the value of these interventions is an important area for future study.

Although penetration of the MyCHOIS-CommonGround application was high in our two pilot clinics, this study suggests that clinic implementation factors had an impact on CommonGround use by clinic clients. We used a well-established implementation framework, the CFIR, to explore differences in clinic implementation to provide context to the divergent adoption profiles of the two pilot sites (Damschroder et al. 2009). Consistent with this framework, we found that Clinic 1 had greater implementation challenges in every CFIR domain, and lower use of the MyCHOIS-CommonGround shared decision-making application by their clinic patients, in both the proportion of patients completing shared decision-making reports and the number of reports completed among these users.

One striking finding was the impact of CFIR implementation process domain, particularly the process of embedding the program within routine clinical workflow. This was illustrated by the rapid increase in Clinic 1 MyCHOIS-CommonGround user profiles (from 36 to 74%) after integrating this step into existing clinical workflow. The change to an embedded approach after 1 year of implementation yielded a penetration in Clinic 1 comparable to Clinic 2 where an embedded approach had been used from the outset. These findings provide a case example of the impact of embedding innovations within organizational procedures and clinical workflow (Aarons et al. 2014; May 2006) and mirrors a previously published study of CommonGround in which the authors attribute program closure to lack of full integration (Bonfils et al. 2016).

The implementation process at each clinic was based on the decisions of local leadership and reflected their response to challenges in all five domains of the CFIR. Clinic directors have a critical role in the success of a MyCHOIS-CommonGround implementation. A recent review by Aarons et al. highlights how leaders’ choices, such as decisions related to resource allocation and integration of new practices within clinic procedures, can create a strategic climate supporting implementation (Aarons et al. 2014). We found that the two clinic leaders differed in their commitment to the project, in part due to differences in their autonomy in the implementation decision. In Clinic 2 the director made the decision to implement, and her subsequent decisions communicated a commitment to the program’s success and created a climate favorable to implementation. Alternatively, Clinic 1’s director did not have the autonomy to make the implementation decision; in managing the project her choices reflected concerns about minimizing the resource burden of the pilot program, inadvertently creating a climate that did not favor implementation. Our study underscores the importance of engaging implementation leaders, particularly middle managers who did not make the implementation decision but are responsible for its execution (Guth and Macmillan 1986). In addition, it highlights that a leadership decision both communicate the importance and prioritization of the new program and determine whether the program will have the necessary resources and infrastructure support to succeed (Aarons et al. 2014; Spetz et al. 2012).

Within the inner setting, staff attitudes toward shared decision-making impacted implementation at each site. Previous research on shared decision-making indicates that enthusiasm and positive attitudes of clinicians are essential in engaging patients (Légaré and Thompson-Leduc 2014). One weakness in our implementation protocol was that little time was spent on the engaging physicians prior to implementation, and they were ambivalent about the shared decision-making program. However, in Clinic 2, where patient use was high and peer support staff stable, a physician reported that despite initial ambivalence he came to depend upon the MyCHOIS-CommonGround reports over time and began to ask all his patients to complete a report prior to their appointment. This suggests a reciprocal and synergistic relationship between patient and physician engagement, where engaged patients can help engage their physicians, and engaged physicians can support patient engagement in shared decision-making.

Outer setting, specifically lack of reimbursement for peer staff supporting the shared decision-making program, was a challenge for both clinics. Our findings support previous reports on the importance of dedicated resources and non-physician support staff in the implementation of new technologies (Ludwick and Doucette 2009; Spetz et al. 2012). Our implementation protocol depended upon peer staff to support patient login, computer use, printing, and review of shared decision-making reports in preparation for physicians’ visits. Consequently, when peer staff were not available, few to no shared decision reports were generated. Intermittent suspension of the program disrupted the process of normalizing the ongoing use of the program for patients and physicians (May 2006). Use of externally funded peer staff, as in Clinic 1, engendered role confusion and challenges in effectively integrating and retaining these peer staff within the clinic. This finding is consistent with previous reports that identify role clarity as an important facet of organizational climate and effective implementation (Aarons et al. 2012; Chinman et al. 2008; Gates et al. 2010; Innis et al. 2015).

The dependence of the program on peer support staff is a challenge for program expansion and sustainability, given that shared decision-making and clinic-based peer staff services are not generally reimbursable or incentivized by insurance plans. There are limited examples, within a few states, of managed care plans providing enhanced reimbursement for physician’s visits that include shared decision-making. One managed care plan received a Gold Award from the American Psychiatric Association for supporting the implementation of the CommonGround shared decision-making program in 12 mental health programs (Schuster et al. 2013). Further study on the impact of the CommonGround shared decision-making program for individuals with serious mental illness is underway (MacDonald-Wilson 2013), and will help policy makers and health care plans to make informed decisions about incentivizing the delivery of these services. In addition, future study is needed to determine whether other methods of supporting use of HIT tools by individuals in specialty mental health services can achieve similar levels of penetration.

Results of this evaluation may not be generalizable to other settings given the voluntary nature of clinics’ participation in the pilot implementation and the small number of participating clinics. Additional research is needed to determine if high levels of penetration can be achieved in a larger sample of clinics serving individuals with serious mental illness. Measures of use were calculated as a proportion of clinic census, which may underestimate penetration due to patient turnover. Although this paper focuses on clinic implementation and the impact on client use, it is also likely that client characteristics impact use. Client data on age, education level, and other characteristics that may impact use is lacking, and an important limitation of the paper. Examination of the relationship between client characteristics and use of web-based applications is an important area for future study.

## Conclusion

This study demonstrates that most individuals receiving specialty mental health services can participate in a web-based shared decision-making program in routine treatment settings, given sufficient support. In addition, it provides evidence that how mental health clinics implement computer-based programs matters and can influence patient adoption.
